# Fast Nearly ML Estimation of Doppler Frequency in GNSS Signal Acquisition Process

**DOI:** 10.3390/s130505649

**Published:** 2013-04-29

**Authors:** Xinhua Tang, Emanuela Falletti, Letizia Lo Presti

**Affiliations:** 1 NavSAS Group, Department of Electronics and Telecommunications, Politecnico di Torino, Corso Duca degli Abruzzi 24, Torino 10129, Italy; E-Mail: letizia.lopresti@polito.it; 2 NavSAS Group, Istituto Superiore Mario Boella, Via Pier Carlo Boggio 61, Torino 10138, Italy; E-Mail: falletti@ismb.it

**Keywords:** acquisition, CAF, refinement, CRLB, least square, averaging method

## Abstract

It is known that signal acquisition in Global Navigation Satellite System (GNSS) field provides a rough maximum-likelihood (ML) estimate based on a peak search in a two-dimensional grid. In this paper, the theoretical mathematical expression of the cross-ambiguity function (CAF) is exploited to analyze the grid and improve the accuracy of the frequency estimate. Based on the simple equation derived from this mathematical expression of the CAF, a family of novel algorithms is proposed to refine the Doppler frequency estimate with respect to that provided by a conventional acquisition method. In an ideal scenario where there is no noise and other nuisances, the frequency estimation error can be theoretically reduced to zero. On the other hand, in the presence of noise, the new algorithm almost reaches the Cramer-Rao Lower Bound (CRLB) which is derived as benchmark. For comparison, a least-square (LS) method is proposed. It is shown that the proposed solution achieves the same performance of LS, but requires a dramatically reduced computational burden. An averaging method is proposed to mitigate the influence of noise, especially when signal-to-noise ratio (SNR) is low. Finally, the influence of the grid resolution in the search space is analyzed in both time and frequency domains.

## 1. Introduction

The main purpose of the acquisition and tracking systems of a Global Navigation Satellite System (GNSS) receiver is to provide an estimate of the Doppler frequency *ƒ_d_*, the code delay τ, and the phase of the carrier, *ϕ*, of the signal transmitted by each visible satellite. The task of the acquisition system is to detect the visible satellites and to provide, for each detected satellite, a coarse estimate $\langle {\hat{f}}_{d}^{a},{\hat{\tau }}^{a}\rangle$ of *ƒ_d_* and τ. This parameter vector is then passed to the tracking systems, whose task is to refine this estimate. The refinement of Doppler frequency estimate is generally performed by a classic phase lock loop (PLL), which requires an initial estimate much more accurate than the one provided by the acquisition system. Therefore it is necessary to improve the accuracy of the estimate ${\hat{f}}_{d}^{a}$ to an acceptable level before starting the operations of the phase tracking loop. A system typically adopted by a GNSS receiver to reach this goal is a frequency lock loop (FLL), which is generally integrated within a PLL. The first refinement is done by a robust FLL operating at wideband, then the loop bandwidth is gradually reduced and finally the system switches to a PLL scheme [1,2]. Other methods [3,4] refine the frequency estimate by exploiting the phase difference between two successive periods of data. An interpolation method is introduced in Reference [5] to estimate the true value of the Doppler frequency, but it is based on an empirical approximation.

In most of the previous methods, usually the estimates of *ƒ_d_* and τ are picked in a search plane only considering the peak cell without any usage of the other cells. In the fields of communications, audio, medical, instrumentation, and others [6], the problem of estimating the frequency of a tone contaminated with noise is tackled for example by Quinn [7,8], MacLeod [9,10], and Jacobsen [6,11], by exploiting the idea of refining the final frequency estimate using the peak sample and two neighbors of the discrete Fourier components. At the same time there are other methods, studied in Reference [12,13], which utilize the phase information. These methods cannot be directly applied to the acquisition of a GNSS signal, because of the very low signal-to-noise ratio and the different signal model, but they can inspire us to do some innovation in GNSS frequency estimation.

In this paper, the peak and neighbor points of the cross-ambiguity function (CAF) in the frequency domain are used to derive a simple formula that greatly improves the accuracy of the frequency estimate provided by the acquisition system. The CAF was initially derived in Reference [14] using statistical principles, then Reference [15] presented a new approach of the CAF derivation. Furthermore in this paper the approximation in CAF main lobe is analyzed in details, based on this approximation, and a new family of methods for refining the estimate of the Doppler frequency is proposed, which exploits the cells close to the peak in the search plane. Compared with the traditional methods, these methods significantly improve the accuracy without increasing the computation complexity or using additional received data.

A preliminary version of this work was presented in Reference [16]. With respect to that previous work, here we extend and complete all the mathematical derivations, extend the performance analysis with appropriate comparisons, derive and discuss the Cramer-Rao lower bound (CRLB) for the frequency estimator showing that the proposed approach is close to the CRLB (quasi-ML approach), and include the theoretical analysis of other non-AWGN nuisances.

This paper is organized as follows. In Section 2 the signal model is presented and the approximate mathematical expression of the CAF in the main lobe is obtained. In Section 3 a new family of algorithms is derived and proved to work perfectly in the absence of noise. In Section 4 the performance of the proposed algorithms is investigated in the presence of additive noise; both the CRLB and a least-square (LS) solution are derived as benchmark, and the comparison shows that the new algorithms can approach very closely the CRLB. Besides that, a simple averaging approach based on non-coherent sums is proposed to improve the accuracy of the algorithms in low SNR conditions. Furthermore, in Section 5, the effects of other nuisances, uncorrelated with the additive noise, are analyzed, and some countermeasures are proposed. Finally, in Section 6 the conclusion is drawn.

## 2. Fundamentals of the New Algorithms

**Figure 1.**
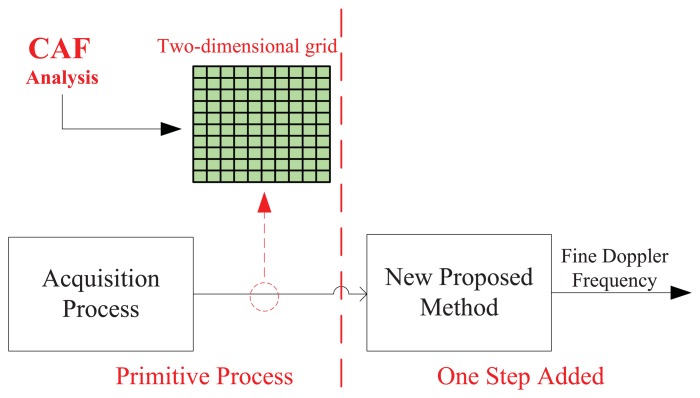
Brief structure of new Doppler frequency refinement process.

The basic scheme of the acquisition method proposed in this paper is illustrated in Figure 1. The left part of the figure indicates the traditional GNSS acquisition process from which a two-dimensional search grid (marked in green color) is generally obtained, while the right side shows the presence of a new additional block able to refine the Doppler frequency estimate in a simple way. The innovation proposed in this paper refers to the algorithms used by this additional block to refine the frequency estimate.

### 2.1. Acquisition Process

The acquisition system for GNSS application is based on the maximum-likelihood estimation theory, which can be briefly described as follows [17].

The incoming sampled signal can be denoted as a vector

(1) y=[y(0)y(1)⋅⋅⋅y(L−1)]

where *L* is the total number of the samples, and

(2) y(n)=r(n)+W(n)

where *r*(*n*) is a signal that contains a vector of unknown parameters a = [*α*_1_*α*_2_*α*_3_ ⋯ *α_K_*], *W*(*n*) is a zero-mean White Gaussian Noise (WGN) random process with variance *σ^2^*, and 0 ≤ *n* ≤ *L* — 1.

The ML estimate of the parameter vector a can be found by maximizing the likelihood function, which depends on the probability density function (PDF), that is

(3) $$\begin{array}{cc} p\left(\text{y};\bar{\text{a}}\right) & =\frac{1}{{\left(2\pi \sigma^{2}\right)}^{\frac{L}{2}}}\exp\left[-\frac{1}{2\sigma^{2}}\sum_{n=0}^{L-1}{\left(y\left(n\right)-\bar{r}\left(n\right)\right)}^{2}\right]\\ & =\frac{1}{{\left(2\pi \sigma^{2}\right)}^{\frac{L}{2}}}\exp\left[-\frac{1}{2\sigma^{2}}\sum_{n=0}^{L-1}\left(y^{2}\left(n\right)+{\bar{r}}^{2}\left(n\right)\right)+\frac{1}{2\sigma^{2}}\sum_{n=0}^{L-1}y\left(n\right)\bar{r}\left(n\right)\right] \end{array}$$

where the test signal *r̅*(*n*) has the same structure of *r*(*n*), but the unknown parameter vector a is substituted by a vector a̅, whose elements are variables defined in a range *ran*_a_ that contains all the possible values of the unknown vector a, that is to say, *a̅* ϵ *ran*_a_.

If the energy of the test signal *r̅*(*n*) (that is the term $\overset{L-1}{\underset{n=0}{\text{∑}}}{\bar{r}}^{2}(n)$ in Equation (3)) does not depend on a̅, then it is possible to show [17] that the corresponding ML estimate â*_ML_* of a is

(4) $${\hat{\text{a}}}_{\mathrm{ml}}=\arg\underset{\bar{\text{a}}\in {\text{ran}}_{\text{a}}}{\max}\sum_{n=0}^{L-1}y\left(n\right)\bar{r}\left(n\right)$$

So, in this case, the ML estimation actually depends on the scalar product *R*(*a̅*) between the test signal and the received signal, defined as

(5) $$R\left(\bar{\text{a}}\right)=\sum_{n=0}^{L-1}y(n)\bar{r}(n)$$

â*_ML_* can be found by searching the maximum *R*(*a̅*) in the range *ran*_a_.

In GNSS field, without considering the influence of noise, the received signal, after down-conversion and sampling, can be written as [1]

(6) $$y\left(nT_{s}\right)=\sum_{m=1}^{N_{\upsilon }}y_{m}\left(nT_{s}\right)$$

where *N_υ_* is the number of satellites in view, and

(7) $$y_{m}(nT_{s})=A_{m}C_{m}(nT_{s}-\tau_{m})d_{m}(nT_{s}-\tau_{m})\cos(2\pi (f_{IF}+f_{d,m})nT_{s}+\varphi_{m})$$

where *A_m_* is the amplitude of the signal, *C_m_*(*nT_s_* −*τ_m_*) = *c_m_* (*nT_s_* −*τ_m_*) *sb*(*nT_s_* −*τ_m_*) is the product of the satellite spreading code *c_m_*(*nT_s_* −*τ_m_*) and subcarrier *s_b_*(*nT_s_* −*τ_m_*) used in the new GNSS systems [18], such as in Galileo (if no subcarrier is present, then *s_b_*(*nT_s_* −*τ_m_*)=1), *τ_m_* is the code delay, *d_m_*(*nT_s_* −*τ_m_*) is the navigation data, *f_IF_* is the intermediate frequency, *f_d,m_* is the Doppler frequency shift, *φ_m_* is the phase of the carrier, and *T_s_* is the sampling interval (the inverse of the sampling frequency *f_s_*).

From Equation (7), we can learn that in principle, the satellite signal actually contains four unknown parameters: code delay (*τ*), Doppler frequency (*f_d_*), carrier phase (*φ*) and data bit. However in the acquisition process, only two of them are estimated, which are *τ* and *f_d_*.

With respect to the parameter data bit, in the implementation, a non-coherent acquisition scheme is used to solve the problem, so here we assume that there is no data-transition in the accumulation period.

Considering the parameter carrier phase, its influence can be removed by involving two components in the acquisition process that are in-phase component (I) and 90° phase-shifted quad-phase (Q) component [19]. Therefore, the test signal *r̅*(*n*) can be written as

(8) $$\bar{r}(n)=C(nT_{s}-\bar{\tau })e^{2\pi \left(f_{IF}+{\bar{f}}_{d}\right)nT_{s}}$$

where the parameter vector becomes $\bar{\text{a}}=\left[\bar{\tau },{\bar{f}}_{d}\right]$, and the energy of *r̅*(*n*) is not related to a̅. So the accumulation process in acquisition can be expressed mathematically as

(9) $$R(\bar{\tau },{\bar{f}}_{d})=\frac{1}{L}\sum_{n=0}^{L-1}y(n)C(nT_{s}-\bar{\tau })e^{2\pi \left(f_{IF}+{\bar{f}}_{d}\right)nT_{s}}$$

Equation (9) is known as cross-ambiguity function (CAF). Based on Equations (4) and (5), the ML estimate of [*τ, f_d_*] can be obtained [17], as

(10) $${\hat{\text{a}}}_{\mathrm{ml}}=\arg\underset{{\text{ran}}_{\text{a}}}{\max}\left|R(\bar{\tau },{\bar{f}}_{d})\right|$$

where |·| means the modulus of a complex value, and the range of a̅, *ran*_a_ will be discussed in Section 3.

There are mainly three acquisition schemes [19]: serial search acquisition, parallel frequency space search acquisition and parallel code phase search acquisition. No matter what kind of scheme is used, a two-dimensional search grid (Figure 4) is always obtained, and the resulting estimated vector is selected as the location of the peak cell, and, at the same time, the other cells in the search grid are abandoned. However, because of the large frequency searching step *f_sp_*, the frequency estimate error is located in the range $\left[-\frac{f_{sp}}{2},\frac{f_{sp}}{2}\right]$, so the initial Doppler frequency estimate is usually not accurate enough to pass to the tracking loop directly.

In order to refine the Doppler frequency estimate, a system typically used is the Frequency Lock Loop (FLL) mentioned in Reference [1,2]. An FLL needs additional data and a special structure, which is generally embedded inside the tracking loops. Another typical technique [3,4], which exploits the phase relation of consecutive data (p.150 in Reference [3]), also needs additional data, and, at the same time, encompasses an ambiguity problem in the phase measurement that has to be solved. Actually this technique is essentially similar to an FLL with a particular discriminator.

In this paper, we develop new methods to refine the Doppler frequency estimate, based only on the search grid already evaluated by the acquisition; that is, we do not have to compute new correlations, but we only use the neighbor cells of the CAF peak, already available in the search grid.

### 2.2. Analytical Expression of the CAF

The CAF [15] is used in radar, sonar and other similar systems to estimate the time delay and the Doppler shift of an incoming signal. An accurate estimation of these signal parameters generally requires the evaluation of several CAF samples, at the cost of an increased computational complexity. In this paper we propose a family of methods that exploits the knowledge of an approximate expression of the analytical formula of the CAF, given in Reference [15], to reach a trade-off between accuracy and complexity.

Following the approach presented in Reference [15], the CAF associated to the generic *i*-th satellite code, locally generated for each trial value of code delay *τ̅* and Doppler frequency *f̅_d_*, can be written as

(11) $$S_{i}(\bar{\tau },{\bar{f}}_{d})=\sum_{m=1}^{N_{\upsilon }}R_{m,i}(\bar{\tau },{\bar{f}}_{d})$$

where *R_m,i_*(*τ̅,f̅_d_*) is the contribution to the CAF of the *m*-th signal *y_m_*(*nT_s_*). Its analytical expression is [15]

(12) $$\begin{array}{cc} R_{m,i}(\bar{\tau },{\bar{f}}_{d}) & =\frac{1}{T_{d}}\int_{0}^{T_{d}}y_{m}(t)\cdot \{C_{i}(t-\bar{\tau })e^{j2\pi {\left(f_{IF}+{\bar{f}}_{d}\right)}^{t}}\}dt\\ & =\frac{A_{m}}{2T_{d}}e^{-j\varphi m}\{F\{P_{T_{d}}(t)\}*F\{b_{m,i}(t)\}\}f=\Delta f\\ & =B_{m}\sum_{k=-\infty }^{\infty }a_{k(m,i)}\sin\text{c}\left(\left(\Delta f-\frac{k}{T_{p}}\right)\pi T_{d}\right)e^{-j(\Delta f-\frac{k}{T_{p}})\pi T_{d}} \end{array}$$

where *B_m_* = (*A_m_*/2*T_d_*)*e*^−*jφm*^, the subscript *i* denotes the *i*-th satellite code generated by the local generator, *T_d_* is the integration time, *F*{} denotes Fourier transform, the symbol * denotes convolution operation, *T_p_* is the code period, *τ̅* is the code delay estimate introduced in the local code, Sin*c*(*x*) = sin (*x*)/*x*, *f̅_d_* is the Doppler frequency estimate introduced in the local carrier, Δ*f* is the Doppler frequency estimate error, which can be expressed as

(13) $$\Delta f=f_{d,m}-{\bar{f}}_{d}$$

*b_m_,_i_*(*t*) is the product of two spreading codes, that is

(14) $$b_{m,i}\left(t\right)=C_{m}(t-\tau_{m})C_{i}(t-\bar{\tau })$$

and *P_Td_* is a window function defined as

(15) $$P_{T_{d}}=\{\begin{array}{cc} 1, & 0<t<T_{d}\\ 0, & \text{otherwise} \end{array}$$

Since *b_m,i_*(*t*) is a periodic signal with a period equal to the code period, its Fourier transform leads to a line spectrum with coefficients given by

(16) $$a_{k(m,i)}=\frac{1}{T_{p}}\int_{T_{p}}b_{m,i}(t)e^{-jk(2\pi /T_{p})}dt$$

and the convolution with the line spectrum leads to the summation in Equation (12).

**Figure 2.**
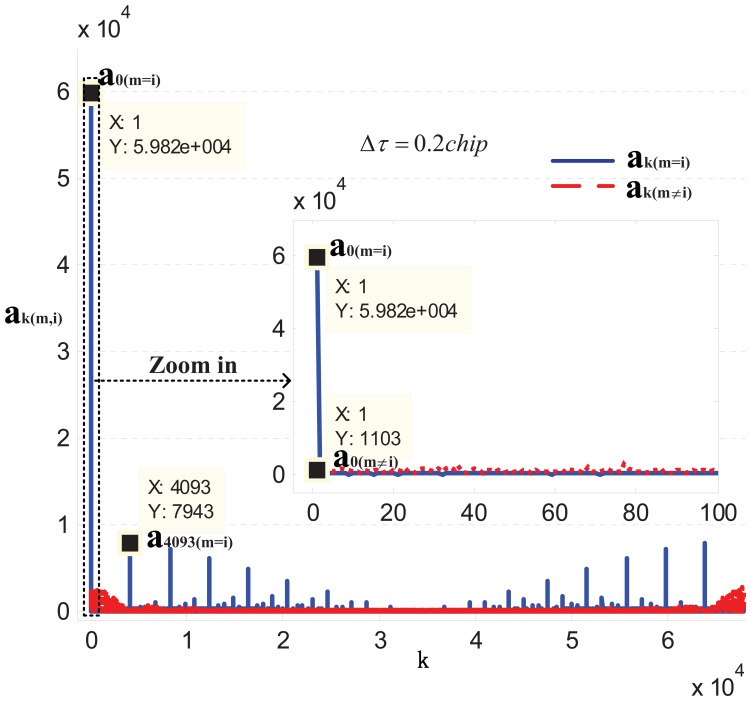
The value of *a_k_* when $\tau_{m}-\bar{\tau }=\Delta \tau =0.2T_{ch}$ in the GPS case.

**Figure 3.**
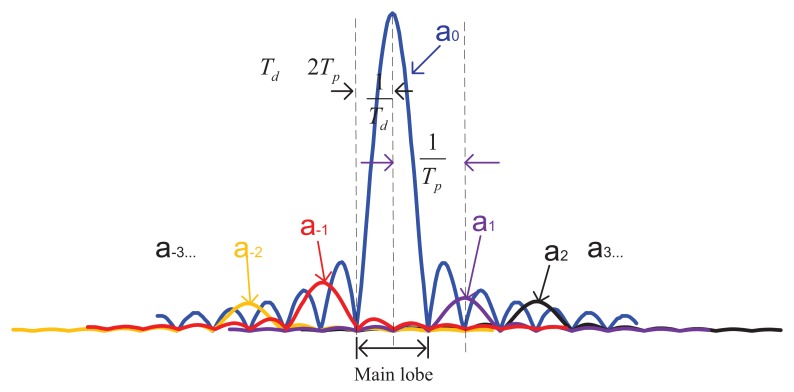
The combination of sinc functions.

In Figure 2 the distribution of *a_k(m,i)_* is shown in the case *τ_m_* −*r̅* = 0.2 *T_ch_*, where *T_ch_* is the chip duration. Thanks to the property of Pseudo Random Noise (PRN) code, as expected, *a_0(m_*_=_*_i)_* predominates over the other *a_k(m,i)_*. To better understand the nature of the summation in Equation (12), let us refer to Figure 3 where for simplicity, we represent the quantity

(17) $$\sum_{k=-\infty }^{\infty }a_{k(i,i)}\left|\text{Sinc}\left(\left(\Delta f-\frac{k}{T_{p}}\right)\pi T_{d}\right)\right|$$

to demonstrate the relationship among different Sinc functions. As Figure 3 shows, if *a*_0_ is the peak component in Equation (12) (the subscript *i,i is* omitted to simplify the notations) only the components strictly adjacent to *a*_0_(*i.e.*, *a*_-2_*a*_-1_*a*_1_*a*_2_ …) affect the shape of the main lobe of Equation (12), while the contribution of faraway components can be ignored. So, if we can guarantee that the adjacent coefficients (*a*_-2_*a*_-1_*a*_1_*a*_2_ …) are far smaller than α_0_, the mathematical expression of the CAF in the main lobe (subscript “ml”) can be written as

(18) $$\begin{array}{cc} S_{i}{\left(\bar{\tau },{\bar{f}}_{d}\right)}_{\text{ml}} & =R_{i,i}{\left(\bar{\tau },{\bar{f}}_{d}\right)}_{\mathrm{ml}}+\sum_{m\neq i}R_{m,i}{\left(\bar{\tau },{\bar{f}}_{d}\right)}_{\mathrm{ml}}\\ & \approx \frac{A_{i}}{2}e^{-\varphi_{i}}a_{0}\text{sinc}(\Delta f\pi {\text{T}}_{d})e^{-\text{j}\Delta f\pi T_{d}} \end{array}$$

where *α*_0_ = α_0(_*_i,i_*_)_ = R(Δ*τ*), and Δ*τ* = *τ*_*m*_ − *τ̅*. As a conclusion the approximate expression of the CAF in its main lobe is

(19) $$S_{i}{\left(\bar{\tau },{\bar{f}}_{d}\right)}_{\text{ml}}\approx \frac{A_{i}}{2}e^{-j\varphi_{i}}R(\Delta \tau )\text{Sinc}(\Delta f\pi T_{d})e^{-j\Delta f\pi T_{d}}$$

The validity of this approximation can be improved in two ways:

1. By enlarging the integration time *T_d_*. In fact as *T_d_* increases, the adjacent components will “move” away relatively In other words, as *T_d_* increases the width of the lobe decreases, while the distance between two sinc functions stays constant, as it depends on the code period.
2. By decreasing the values of the adjacent coefficients (*a*_−2_*a*_−1_*a*_1_*a*_2_ …). This can be obtained by improving the accuracy of code delay estimate, so as to work close to the maximum of *R*(Δ*τ*).

Based on the CAF expression in Equation (19), new algorithms for a better estimation of the Doppler frequency are discussed hereafter, both in ideal (*i.e.*, noiseless, Section 3) and realistic (Section 4) scenarios.

## 3. Doppler Frequency Evaluation in the Absence of Noise

**Figure 4.**
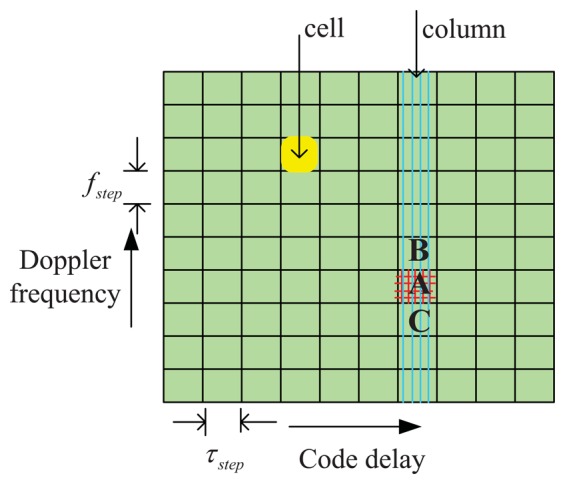
Two-dimensional search space.

The signal acquisition process is basically a two dimensional search in a grid plane (commonly referred to as *search space*), as shown in Figure 4, where *τ̅ ϵ* (0, *T_p_*) (X-axis range), and *f̅_d_* ∈ (−*f_dmax_*, *f_dmax_*) (Y-axis range). The variables under test *τ̅* and *f̅_d_* are discretized with a step *τ_sp_* for the code delay, and a step *f_sp_* for the Doppler frequency. The integration time is *T_d_* = *LT_s_* (where *L* is the total number of integrated samples). The number of trial points in the two axes are *N_τ_* = *T_d_/τ_sp_*, and *N_f_* = 2*f_dmax_/f_sp_*. Therefore the grid plane contains *N_τ_* × *N_f_* cells, and each cell (marked by yellow color in Figure 4) corresponds to a parameter pair 〈*τ̅*, *f̅_d_*〉. Finally the decision variable for the acquisition is

(20) $$\bar{S}{\left(\bar{\tau },{\bar{f}}_{d}\right)}_{\text{ml}}=\left|S_{i}{\left(\bar{\tau },{\bar{f}}_{d}\right)}_{\text{ml}}\right|$$

The purpose of a traditional acquisition system is to find the coordinates of the peak cell of the grid plane when the satellite we want to detect is visible. To improve the accuracy of the estimates, the steps *τ_sp_* and *f_sp_* must be decreased, at the expenses of the computational complexity, since the number of points of the search space increases. The empirical value *f_sp_* = 2/(3*LT_s_*) is a typical choice [8] for the Doppler frequency step.

**Figure 5.**
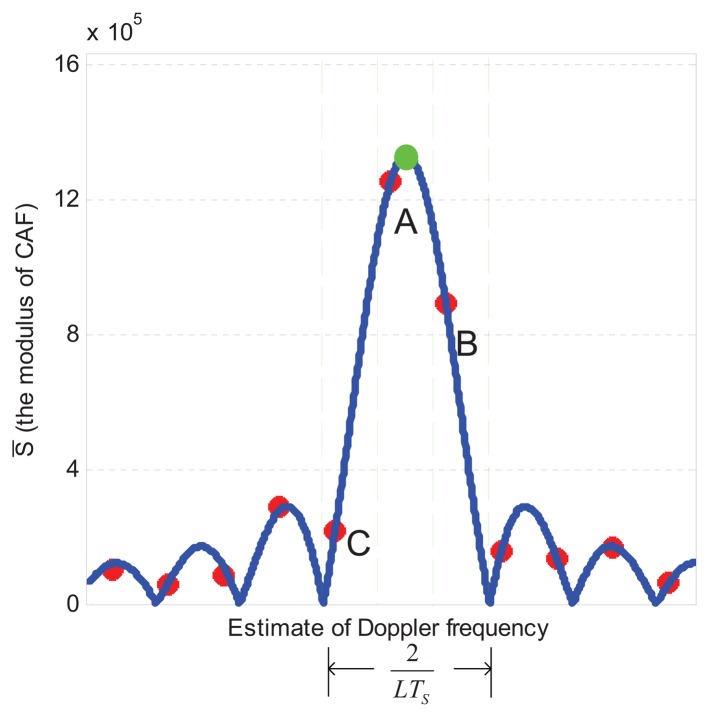
The plot of the column (*τ̅*≈*τ*)

An example of search space is shown in Figure 4. The column (marked in green) crossing the peak cell (marked A) contains cells that share the same code delay. When the *i*-th satellite is visible, the function in this column is as shown in Figure 5, where the X-axis contains the variable Doppler frequency *f̅_d_*, while the Y-axis represents the absolute value of the acquisition test statistic *S̅*(*τ̅*, *f̅_d_*)_ml_. As indicated in Figure 3, the width of the main lobe is 2/*T_d_* = 2/(*LT_s_*). Since *f_sp_* = 2/(3*LT_s_*), this guarantees that three adjacent points of the frequency domain CAF (A, B, C) are located in the main lobe, and then we can assume that Equation (19) is always valid in these points.

The cells A, B and C are characterized by the triplete and (*τ̅_A_,f̅_A_S̅*_a_),(*τ̅_B_,f̅_B_S̅_B_*),and(*τ̅_C_,f̅_C_S̅_C_*), where the code delay is the same for all the cells in the same column (*τ̅*_a_=*τ̅_B_τ̅_C_*) and we adopt the notation *S̅_X_* = *S̅*(*τ̅_X_*, *f̅_dX_*) *_ml_*, *X* = (*A,B,C*) for simplicity. In the case of no noise, these triplets can be used to find the true Doppler frequency *f_d_*, as it will be shown hereafter. This will be also the starting point of the estimation method proposed in this paper, when the measurements are affected by noise.

### 3.1. True Solution Based on the Absolute Value of CAF

In Reference [16], which is our initial work on this topic, we can also find the basic idea about the solution based on the absolute value of the CAF. Under the assumption that there is no data transition in the integration interval, based on Equations (19) and (20) we can write the following equations [16]

(21) $$\begin{array}{cc} {\bar{S}}_{A} & =\frac{A}{2}\left|R(\Delta \tau )\right|\left|\text{Sinc}(\pi {\text{LT}}_{\text{s}}({\text{f}}_{\text{d}}-{\bar{f}}_{A}))\right|\\ {\bar{S}}_{B} & =\frac{A}{2}\left|R(\Delta \tau )\right|\left|\text{Sinc}(\pi LT_{s}(f_{d}-{\bar{f}}_{B}))\right|\\ {\bar{S}}_{C} & =\frac{A}{2}\left|R(\Delta \tau )\right|\left|\text{Sinc}(\pi LT_{s}(f_{d}-{\bar{f}}_{C}))\right| \end{array}$$

where the unknowns are the amplitude *A*, the value of the code correlation function *R*(Δ*τ*), and the true Doppler frequency *f_d_*. We are going to show that the value of *f_d_* can be easily computed from the above system of equations [16].

First of all we observe that, when the points are located in the main lobe (like points A, B and C in Figure 5), we can write |Sinc (*πLT_s_*(*f_d_* − *f̅_A_*))| = Sinc(*πLT_s_*(*f_d_* − *f̅_A_*)). Now, according to Equation (13) we write *f̅* = *f_d_* − Δ*f*, so

(22) $$\begin{array}{ll} \bar{f}\bar{S} & =(f_{d}-\Delta f)\bar{S}\\ & =f_{d}\bar{S}-\frac{A\left|R(\Delta \tau )\right|\sin(\pi LT_{s}\Delta f)}{2\pi LT_{s}} \end{array}$$

Based on Equations (21) and (22), we can write

(23) $${\bar{f}}_{A}{\bar{S}}_{A}+{\bar{f}}_{B}{\bar{S}}_{B}+{\bar{f}}_{C}{\bar{S}}_{C}={\bar{f}}_{d}{\bar{S}}_{A}+{\bar{f}}_{d}{\bar{S}}_{B}+{\bar{f}}_{d}{\bar{S}}_{C}-S_{A,B,C}$$

where

(24) $$S_{A,B,C}=\frac{A\left|R(\Delta \tau )\right|}{2\pi LT_{s}}(\sin(\pi LT_{s}\Delta f_{A})+(\sin(\pi LT_{s}\Delta f_{B})+(\sin(\pi LT_{s}\Delta f_{C}))$$

Considering *f_sp_* = 2/(3*LT_S_*):

(25) $$\begin{array}{lll} {\bar{f}}_{B} & ={\bar{f}}_{A}+\frac{2}{3LT_{s}} & \Rightarrow \Delta f_{B}=\Delta f_{A}+\frac{2}{3LT_{s}}\\ {\bar{f}}_{C} & ={\bar{f}}_{A}+\frac{2}{3LT_{s}} & \Rightarrow \Delta f_{C}=\Delta f_{A}+\frac{2}{3LT_{s}} \end{array}$$

from which

(26) $$S_{A,B,C}=\sin\left(\pi LT_{s}\Delta f_{A}+\frac{2\pi }{3}\right)+\sin\left(\pi LT_{s}\Delta f_{A}-\frac{2\pi }{3}\right)=0$$

By substituting Equation (26) for *S_A,B,C_* in Equation (23) we obtain *f̅_A_S̅_A_* + *f̅_B_S̅_B_* + *f̅_C_S̅_C_* = *f_d_*(*S̅_A_* + *S̅_B_* + *S̅_C_*), from which we can write the true Doppler frequency as

(27) $$f_{d}=\frac{{\bar{f}}_{A}{\bar{S}}_{A}+{\bar{f}}_{B}{\bar{S}}_{B}+{\bar{f}}_{C}{\bar{S}}_{C}}{{\bar{S}}_{A}+{\bar{S}}_{B}+{\bar{S}}_{C}}$$

This expression gives the correct value of the Doppler frequency *f_d_*, independently from the code delay error Δ*τ*, in the absence of noise and other nuisances. This equation represents a weighted average of the three measured points, and can be used as a first promising estimator of the Doppler frequency even when the CAF is affected by noise. Notice that Equation (27) is valid only when the Doppler frequency step is *f_sp_* = 2/(3*LT_S_*).

**Figure 6.**
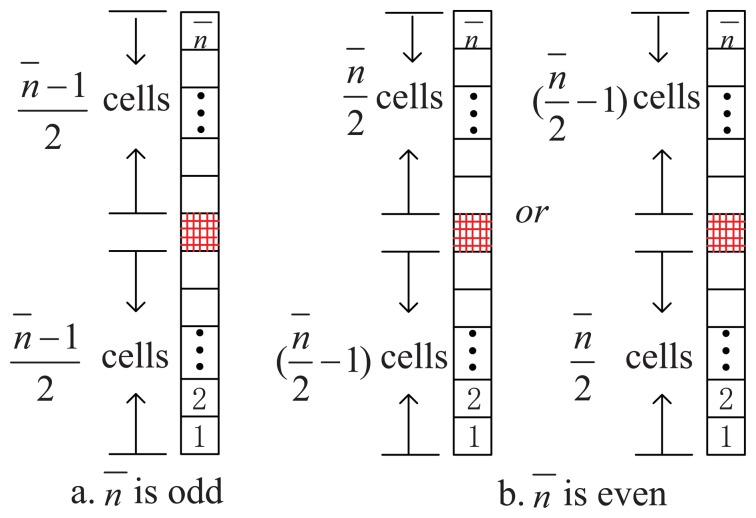
The cells chosen in the generalized method.

More in general, if we choose the Doppler frequency step as

(28) $$f_{sp}=\frac{2}{\bar{n}LT_{s}}$$

where *n̅* is a positive integer, we can choose the cells as follows:

- When *n̅* is odd, we take (*n̅* − 1)/2 cells at each side of the peak cell, as shown in Figure 6(a).
- When *n̅* is even, we take *n̅/2* cells at one side and [(*n̅/2*) − 1]cells on the other side of the peak cell, as Figure 6(b) shows.

No matter what value *n̅* assumes (even or odd), there will be *n̅* cells taken into the final calculation, and Equation (26) will become

(29) $$\sum_{k=0}^{\bar{n}-1}\sin\left(\pi LT_{s}\Delta f_{1}+\frac{2k}{\bar{L}T_{s}}\right)=0$$

where Δ*f*1=*f*_d_−*f̅*_1_ and *f̅*_1_ is the trial value of the Doppler frequency in the first cell of the set (*i.e.*, the cell 1 marked in Figure 6). Then Equation (27) becomes

(30) $$f_{d}=\frac{\overset{\bar{n}}{\underset{k=1}{\text{∑}}}{\bar{f}}_{k}{\bar{S}}_{k}}{\sum_{k=1}^{\bar{n}}{\bar{S}}_{k}}$$

which can be adopted as an estimator of the Doppler frequency in the presence of noise.

For simplicity, hereafter we refer to solution Equation (27) as algorithm R-3 and solution Equation (30) as algorithm R-*n̅*.

### 3.2. True Solution Based on Complex Values of CAF

Based on References [7,10], we can obtain another similar solution by using the test statistic *S̅*(*r̅*,*f̅_d_*)_ml_ in the complex form given in Equation (19). In fact it is possible to show that

(31) $$\begin{array}{ll} f_{d,\text{complex}} & =\text{Real}\left\{\frac{{\bar{f}}_{C}S_{C}+{\bar{f}}_{A}S_{A}e^{-j\pi f_{sp}T_{d}}+{\bar{f}}_{B}S_{B}e^{-j\pi f_{sp}T_{d}}}{S_{C}+S_{A}e^{-j\pi f_{sp}T_{d}}+S_{B}e^{-j\pi f_{sp}T_{d}}}\right\}\\ & =\text{Real}\left\{\frac{\left({\bar{f}}_{C}\left|S_{C}\right|+{\bar{f}}_{A}\left|S_{A}\right|+{\bar{f}}_{B}\left|S_{B}\right|\right)e^{-j(\pi \Delta f_{C}T_{d}+\varphi_{i})}}{(\left|S_{C}\right|+\left|S_{A}\right|+\left|S_{B}\right|)e^{-j(\pi \Delta f_{C}T_{d}+\varphi_{i})}}\right\}\\ & =\frac{{\bar{f}}_{C}\left|S_{C}\right|+{\bar{f}}_{A}\left|S_{A}\right|+{\bar{f}}_{B}\left|S_{B}\right|}{\left|S_{C}\right|+\left|S_{A}\right|+\left|S_{B}\right|}\\ & =f_{d} \end{array}$$

At the same time, similarly to Equation (30), we can generalize this solution as

(32) $$f_{d,\text{complex}}=\text{Real}\left\{\frac{\overset{\bar{n}}{\underset{k=1}{\text{∑}}}{\bar{f}}_{k}S_{k}e^{-j(k-1)\pi f_{sp}T_{d}}}{\overset{\bar{n}}{\underset{k=1}{\text{∑}}}S_{k}e^{-j(k-1)\pi f_{sp}T_{d}}}\right\}$$

where *f̅*_k+1_=*f̅_k_*+*f_sp_* and ƒ_1_ is the Doppler frequency value in cell 1 marked in Figure 6.

For simplicity, hereafter we refer to solution Equation (31) as algorithm C-3 and solution Equation (32) as algorithm C-*n̅*.

### 3.3. Test of Validity

The formulas in the previous sections show that, in the absence of noise and other nuisances, the proposed equations are able to evaluate the true Doppler frequency, hence reducing the estimation error range from the traditional (−*f_sp_*/2, *f_sp_*/2) to theoretically zero. A possible residual error can arise due to the fact that the method is based on an approximate formulation of the test statistic Equation (19). Therefore to test its validity, we set up a simulated acquisition campaign, where the Doppler frequency estimation error due to the traditional acquisition method is compared with the residual error introduced by the algorithms R-3, R-*n̅*, C-3, and C-*n̅*. In the simulations, the GNSS signals are generated by using the signal simulator N-FUELS [20], and several instances of a Galileo E1-b signal are obtained. Firstly we tested the methods in an ideal scenario (*i.e.*, noiseless), with parameters *f_IF_* = 4 MHz, *f_s_* = 17 MHz, *τ* = 0.11 ms.

**Figure 7.**
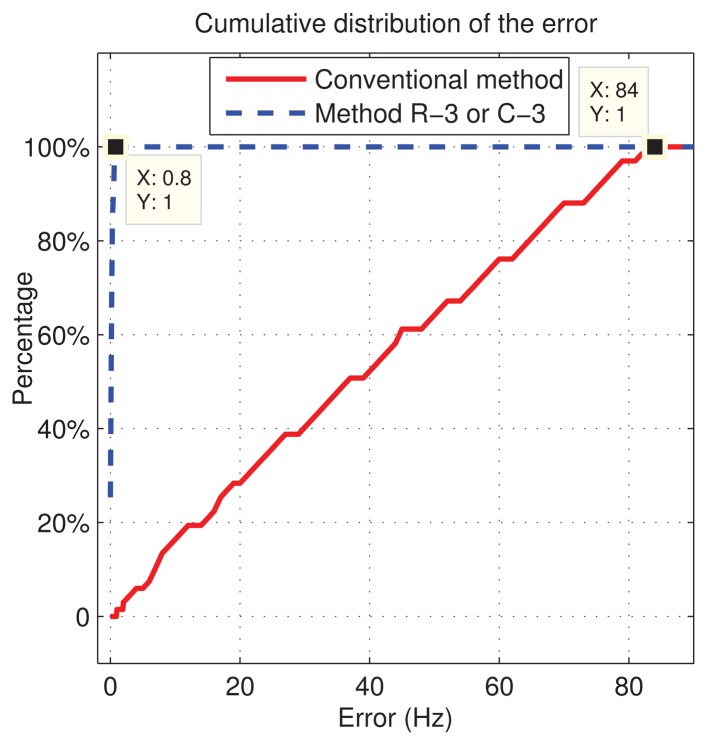
Cumulative distribution of the frequency errors (comparison between the conventional and methods R-3 and C-3).

The accumulation time of the acquisition stage is set to the minimum period (4 ms), assuming that no data transition occurs in this period, the Doppler frequency step is

(33) $$f_{sp}=\frac{2}{3LT_{s}}=167{\text{H}}_{\text{Z}}$$

and 67 different values of *f_d_* are randomly chosen in the range (−4, 500,4, 500) Hz. This makes the original errors uniformly distributed in (−*f_sp_*/2, *f_sp_*/2), as also proved in Figure 7, where the cumulative distribution of the Doppler frequency estimation errors is shown. After the refinements are obtained by using R-3 or C-3, the error range decreases to nearly (−0.8,0.8) Hz, which is a residual numerical error due to the approximations introduced in Equations (18) and (19).

Therefore, we can conclude that in the absence of noise, in experiments, the new methods eliminate the error in the evaluation of the Doppler frequency due to the discretization of the search space, just using three cells selected in the main lobe of the frequency-domain CAF The only constraint is that the Doppler frequency step has to be as given in Equation (28), with *n̅* = 3. Moreover the obtained results show that the method is not affected by the code delay error Δ*τ*.

The practical situation in which the noise is unavoidable is discussed in the next section.

## 4. Doppler Frequency Estimation in the Presence of Noise

In real scenarios, we have to consider the influence of noise, which can be modeled as an Additive White Gaussian Noise (AWGN), as commonly done in the literature. Then the received signal model becomes

(34) $$\begin{array}{ll} \tilde{y}(nT_{s}) & =y(nT_{s})+\eta (nT_{s})\\ & =Ac(nT_{s}-\tau )s_{b}(nT_{s}-\tau )d(nT_{s}-\tau )\\ & \cos(2\pi (f_{IF}+f_{d})nT_{s}+\varphi )+\eta (nT_{s}) \end{array}$$

where *η*(*nT_s_*) represents the white Gaussian noise normally distributed with zero mean and variance $\sigma_{IF}^{2}$, related to the power spectral density *S_N_*(*f*) = *N*_0_/2 of the analogue noise by the well-known formula

(35) $$\sigma_{IF}^{2}=E\{\eta^{2}(nT_{s})\}=\frac{N_{0}f_{s}}{2}$$

valid when the transfer function of the equivalent front-end filter is assumed flat over the whole digitization bandwidth (−*f_s_*/2,*f_s_*/2). Without considering the data-transition, the in-phase and quadrature components of the CAF for the local parameters *f̅_d_* and *τ̅* can be written as

(36) $$\begin{array}{ll} I(\bar{\tau },{\bar{f}}_{d}) & =\frac{1}{N}\sum_{n=0}^{N-1}\left(y(nT_{s})+\eta (nT_{s}))C(nT_{s}-\bar{\tau })\cos(2\pi (f_{IF}+{\bar{f}}_{d})\right)\\ Q(\bar{\tau },{\bar{f}}_{d}) & =\frac{1}{N}\sum_{n=0}^{N-1}\left(y(nT_{s})+\eta (nT_{s}))C(nT_{s}-\bar{\tau })\sin(2\pi (f_{IF}+{\bar{f}}_{d})\right) \end{array}$$

which, based on Equation (19) (and omitting the subscript *i*), becomes

(37) $$\begin{array}{ll} I(\bar{\tau }{\bar{f}}_{d}) & =\text{Real}\left\{S(\bar{\tau },{\bar{f}}_{d})\right\}+N_{I}\\ Q(\bar{\tau }{\bar{f}}_{d}) & =\text{Img}\left\{S\left\{S(\bar{\tau },{\bar{f}}_{d})\right\}+N_{Q}\right\} \end{array}$$

where Real{ } and Img{ } mean respectively real and imaginary part of a complex value, and

(38) $$\begin{array}{ll} N_{I} & =\frac{1}{N}\sum_{n=0}^{N-1}\eta (nT_{s})C(nT_{s}-\bar{\tau })\cos(2\pi (f_{IF}+{\bar{f}}_{d}))\\ N_{Q} & =\frac{1}{N}\sum_{n=0}^{N-1}\eta (nT_{s})C(nT_{s}-\bar{\tau })\sin(2\pi (f_{IF}+{\bar{f}}_{d})) \end{array}$$

According to Reference [21], we know that *N_I_* and *N_q_* are still white noise processes with variance $\mathrm{var}(N_{I})=\mathrm{var}(N_{Q})=\sigma_{IF}^{2}/(2N)$, and the envelope of *S̃*(*τ̅*, *f̅d*) = *I* + *jQ* is

(39) $$\begin{array}{ll} \tilde{\bar{S}}{\left(\bar{\tau },{\bar{f}}_{d}\right)}^{2} & =I{\left(\bar{\tau },{\bar{f}}_{d}\right)}^{2}+Q{\left(\bar{\tau },{\bar{f}}_{d}\right)}^{2}\\ & =\bar{S}{\left(\bar{\tau },{\bar{f}}_{d}\right)}^{2}+2\text{Real}\{S(\bar{\tau },{\bar{f}}_{d})\}N_{I}+2\text{Img}\{S(\bar{\tau },{\bar{f}}_{d})\}N_{Q}+N_{I}^{2}+N_{Q}^{2}\\ & =\bar{S}{\left(\bar{\tau },{\bar{f}}_{d}\right)}^{2}+\omega_{I}+\omega_{Q} \end{array}$$

where the term *w_I_*+*w_q_* = *w* is a random process with mean $E\{w\}=E\{N_{1}^{2}+N_{Q}^{2}\}=\sigma_{IF}^{2}/N$ including all the noise contributions. At this point, working as in Equations (27) or (31), we can develop two new estimators in the presence of noise, that is

(40) $${\hat{f}}_{d}=\frac{{\bar{f}}_{A}{\tilde{\bar{S}}}_{A}+{\bar{f}}_{B}{\tilde{\bar{S}}}_{B}+{\bar{f}}_{C}{\tilde{\bar{S}}}_{C}}{{\tilde{\bar{S}}}_{A}+{\tilde{\bar{S}}}_{B}+{\tilde{\bar{S}}}_{C}}$$

and

(41) $${\hat{f}}_{d,\text{complex}}=\text{Real}\left\{\frac{{\bar{f}}_{C}{\tilde{S}}_{C}+{\bar{f}}_{A}{\tilde{S}}_{A}e^{-j\pi f_{sp}T_{d}}+{\bar{f}}_{B}{\tilde{S}}_{B}e^{-j\pi f_{sp}T_{d}}}{{\tilde{S}}_{C}+{\tilde{S}}_{A}e^{-j\pi f_{sp}T_{d}}+{\tilde{S}}_{B}e^{-j\pi f_{sp}T_{d}}}\right\}$$

(for simplicity the Doppler frequency step is as in Equation (33)).

Hereafter, we refer to Equation (40) as algorithm “R*_n_*-3” and to Equation (41) as algorithm “C*_n_*-3”. As before, they can be generalized to “R*_n_-n̅*” and “C*_n_-n̅*”

In the following two subsections, we discuss two terms of comparison worth to be considered for the frequency estimators proposed so far, firstly a least squares solution and secondly the CRLB on the variance of the estimator. Performance comparisons obtained in simulation are presented in Section 4.3.

### 4.1. Least-Square Method

Another approach to exploit the CAF points ${\tilde{\bar{S}}}_{A},{\tilde{\bar{S}}}_{B},{\tilde{\bar{S}}}_{C}$ as defined in Equation (39) is to set up a least squares (LS) problem as follows.

Since we know that the main lobe of the CAF is a sinc function, it is possible to use the LS method in which the fitting curve is the sinc function Equation (21). The LS method requires that the sum of the squared residuals

(42) $$Q_{LS}(\bar{\text{a}},f_{d})={\left({\tilde{\bar{S}}}_{A}-{\bar{S}}_{A}\right)}^{2}+{\left({\tilde{\bar{S}}}_{B}-{\bar{S}}_{B}\right)}^{2}+{\left({\tilde{\bar{S}}}_{C}-{\bar{S}}_{C}\right)}^{2}$$

is minimized with respect to the unknown parameters, where *A̅* = *A*/(2*R*(Δ_*τ*_)). This leads to the equations

(43) $$\begin{array}{c} \frac{\partial Q_{LS}(\bar{A},f_{d})}{\partial \bar{\text{a}}}=0\\ \frac{\partial Q_{LS}(\bar{\text{a}},f_{d})}{\partial f_{d}}=0 \end{array}$$

Since we are interested in the performance comparison with R*_n_*-3 or C*_n_*-3, we will use a numerical method to solve Equation (43) for *f_d_*.

### 4.2. Evaluation of the Cramer-Rao Lower Bound

Usually, a statistical estimator is characterized by its bias (mean error), variance (mean square error), and the threshold SNR (signal-to-noise ratio) [22]. Hence, here the Cramer-Rao lower bound (CRLB) is proposed as benchmark to compare the performance of different algorithms.

Without considering the data-transition problem and ignoring the influence of the code delay estimation error (which will be discussed in Section 5.2), the received samples Equation (34) can be mathematically expressed as

(44) $$\begin{array}{ll} \tilde{y}(nT_{S}) & =y(nT_{S})+\eta (nT_{S})\\ & =A\cos(2\pi (f_{IF}+f_{d})nT_{s}+\varphi )+\eta (nT_{S}) \end{array}$$

which contains the unknown parameter vector ***θ*** = [*A*, *f_d_*, *φ*]. The corresponding Fisher information matrix **I** for an observation of *N* samples has elements [22]

(45) $${\left[I(\theta )\right]}_{ij}=\frac{1}{\sigma^{2}}\sum_{n=0}^{N-1}\frac{\partial y(n;\theta )}{\partial \theta_{i}}\frac{\partial y(n;\theta )}{\partial \theta_{j}}$$

where *y*(*n*; ***θ***) = *A* cos(2*π*(*f_IF_* + *f_d_*)*nT_s_* + *φ*), and *N* is the number of samples used for the estimation of the unknowns. Therefore the Fisher matrix is

(46) $$I(\theta )=\frac{1}{\sigma_{IF}^{2}}\left[\begin{array}{ccc} \frac{N}{2} & 0 & 0\\ 0 & 2\pi^{2}A^{2}T_{s}^{2}\sum_{n=0}^{N-1}n^{2} & \pi A^{2}T_{s}\sum_{n=0}^{N-1}n\\ 0 & \pi A^{2}T_{s}\sum_{n=0}^{N-1}n & \frac{NA^{2}}{2} \end{array}\right]$$

The CRLB is found as the [*i, i*] element of the inverse of the Fisher matrix:

(47) $$\mathrm{var}(\theta_{i})\geq {\left[{\text{I}}^{-1}(\theta )\right]}_{ii}$$

Therefore the CRLB for the Doppler frequency estimate is

(48) $$\begin{array}{ll} \mathrm{var}({\hat{f}}_{d}) & \geq {\left[{\text{I}}^{-1}(\theta )\right]}_{22}\\ & \geq \frac{12}{{\left(2\pi \right)}^{2}\rho T_{s}^{2}N(N^{2}-1)} \end{array}$$

where $\rho =A^{2}/\left(2\sigma_{IF}^{2}\right).$ As *N* = *f_s_T_d_* ≫ 1, Equation (48) can be written as

(49) $$\begin{array}{ll} \mathrm{var}({\hat{f}}_{d}) & \geq {\left[{\text{I}}^{-1}(\theta )\right]}_{22}\\ & \geq \frac{12}{{\left(2\pi \right)}^{2}\rho f_{s}T_{d}^{3}} \end{array}$$

which is the benchmark for our estimation algorithms.

### 4.3. Simulation Experiments for Performance Assessment

To test the algorithms R*_n_*-3, C*_n_*-3 and LS, we performed several simulation experiments with different values of the carrier-to-noise density ratio (CNR), corresponding to a signal-to-noise ratio *ρ* given by:

(50) $$\text{SNR}=\frac{C}{N_{0}B}$$

where *B* is the one side front-end bandwidth, assumed ideally flat over the whole digitization bandwidth.

The parameters used in the experiments are *f_IF_* = 4 MHz, *f_s_* = 17 MHz, *τ* = 0.11 ms, and *f_sp_* is set as in Equation (33). We use the root-mean-square error (RMSE) computed for the different algorithms as a metric of performance comparison:

(51) $$f_{\text{RMSE}}=\sqrt{E\left\{{\left({\hat{f}}_{d}-f_{d}\right)}^{2}\right\}}$$

where *f̂_d_* is the Doppler frequency estimate, and *E*{·} is the expected value (estimated as a temporal average along the simulation runs). The metric *f_rmse_* is calculated for each algorithm and compared with the square root of CRLB.

**Figure 8.**
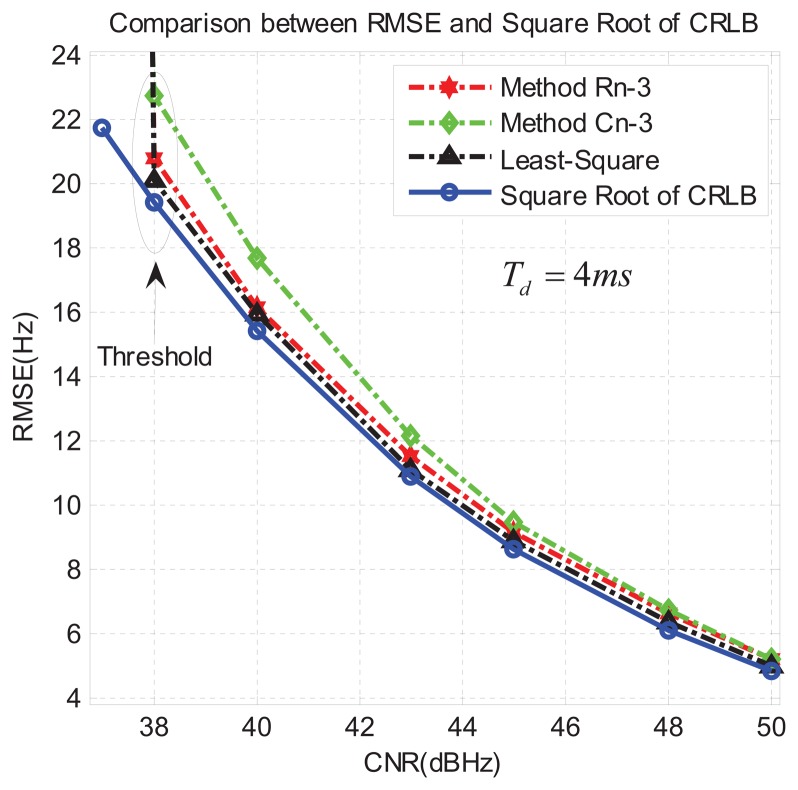
Results of the first group of experiments, with *T_d_* = 4 ms (including the false alarm).

In the first group of experiments, we set *T_d_* = 4 ms and we executed 1000 runs for each CNR. Then, according to Equation (51), we calculated the corresponding RMSE. The results can be seen in Figure 8. We can see that in this case the algorithm R*_n_*-3 is better than C*_n_*-3, as it achieves a lower RMSE closer to the CRLB. At the same time we can see that the algorithm R*_n_*-3 is very close to the least-square method, though the latter is slightly better. However, considering the computation complexity of the LS method, the algorithm R*_n_*-3 appears really competitive with respect to the LS. Finally, the threshold CNR (below which the RMSE rapidly worsens) is around CNR = 38 dB-Hz in all the proposed methods.

**Figure 9.**
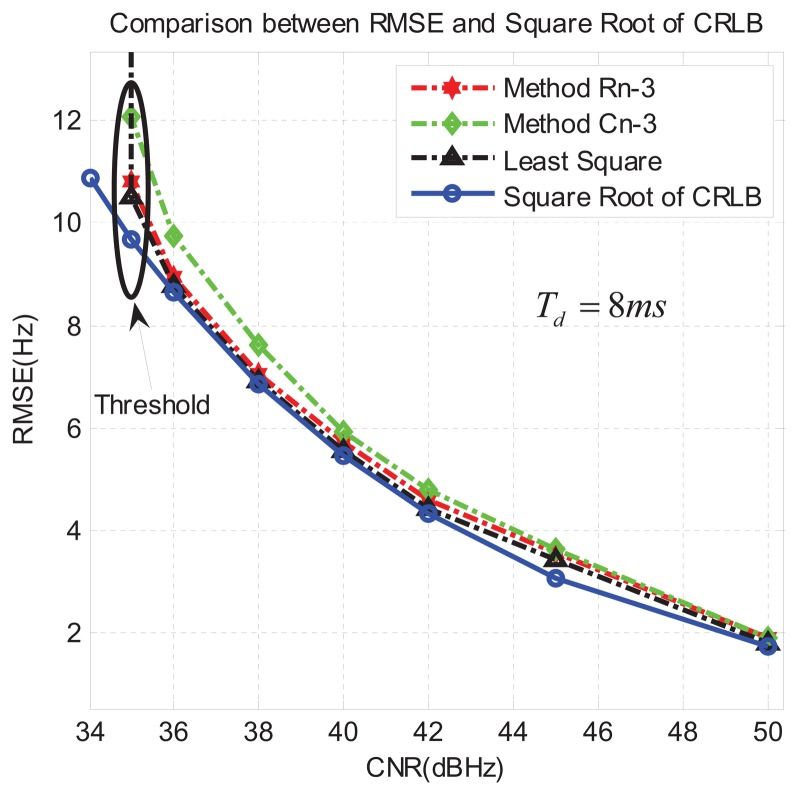
Results of second group of experiments, with *T_d_* = 8 ms. (including the false alarm).

In the second group of experiments, we changed the integration time to *T_d_* = 8ms. Similarly, we executed 1,000 runs for each CNR and obtained the results reported in Figure 9, where we can observe that, as expected, the RMSE is lower than in Figure 8, since a longer integration time reduces the effects of noise. Again, R*_n_*-3 is the closest to the least-square method, and the threshold CNR decreases to around CNR = 35 dB-Hz in all three proposed methods.

Comparing Figures 8 and 9 we can observe that, first, at the same CNR, the RMSE in Figure 9 is decreased by about a factor of 2.8 with respect to Figure 8. This is in agreement with the theoretical CRLB given in Equation (49). In fact when *T_d_* = 4 ms is replaced with *T_d_* = 8 ms, the CRLB bound decreases by a factor of $\sqrt{8}$. Furthermore, when CNR is relatively high (like CNR≥ 45dB-Hz in Figure 8), the proposed three algorithms are very close to the CRLB and each other.

### 4.4. Averaging Method

**Figure 10.**
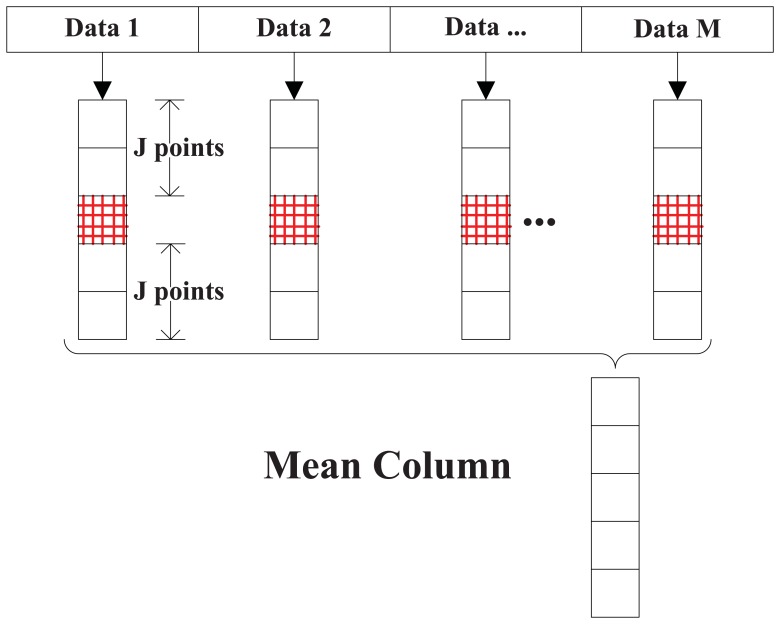
Averaging method.

Since noise is dominant in the acquisition process, in order to increase the robustness of the proposed approach in the presence of noise, the performance of a simple averaging method, based on the idea of non-coherent sums, is assessed here. The main steps of the method represented in Figure 10 are:

1. Find the initial code delay using a first period of data, then pick out the column (marked in blue color in Figure 4), and save (2*J* + 1) points (*J* points at each side of the peak point as illustrated in Figure 10)
2. Use the parameter 〈*f̅_d_*, *τ̅*〉 evaluated in the first step to calculate the new columns (as shown in Figure 10) of the next (*M* − 1) periods of data.
3. Calculate the average of the *M* columns into one single mean column.
4. Pick out the top *n̅* cells in the mean column and use Equation (40) to calculate the final Doppler frequency.

**Figure 11.**
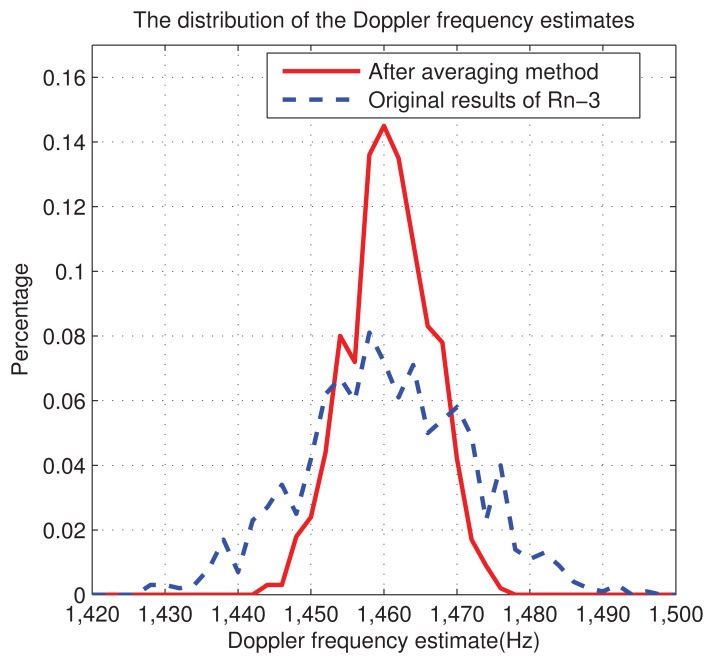
The distribution of the Doppler frequency estimates.

In the following simulation, we set CNR = 43 dB-Hz, *n̅*=3, *J* = 2, *M* = 4, and we execute 1,000 independent runs, for both averaging and non-averaging strategies. The result is shown in Figure 11, where we can see that the new averaging method decreases the error range from (−25,25) Hz to nearly (−15,15) Hz.

## 5. Analysis of Other Non-AWGN Nuisances

The performance of the algorithms presented so far depends not only on the additive noise but also on other nuisances, whose impact is analyzed in this section. In particular we observe that the methods R*_n_*-3 and C*_n_*-3 are based on the two measured vectors f = [*f̅_C_*, *f̅_A_*, *f̅_B_*] and **S̅** = [*S_C_*, *S_A_*, *S_B_*] used in Equations (27) and (31), obtained by reading the peak cell and the adjacent cells in the same column (marked as C, A and B in Figure 4). In particular, the code delay *τ̅_p_* is kept constant in these three cells.

Since the search space is discretized, in general even in the absence of noise *f̅_p_* ≠ *f_d_*, and *τ̅_p_* ≠ *τ*. This can be seen as a quantization error Δ *f_q_* = *f̅_p_* −*f_d_* in the frequency domain, and Δ *τ_q_* = *τ̅_p_* − *τ* in the time domain. We know that Equations (27) and (31) completely eliminate the quantization errors in the absence of noise. However, in the presence of noise, we experience an accuracy degradation due to the noise influence on vector **S̅**, as shown in the simulation results of the previous section. The purpose of the following analysis is to state the influence of the frequency and code delay quantization errors on the proposed frequency estimators, in the presence of noise.

### 5.1. The Influence of the Peak Point's Location

In this section we study the influence of the quantization error in the frequency domain, which can be re-elaborated as

(52) $$\Delta_{p}=\left|\frac{\Delta fq}{f_{sp}}\right|$$

from which it is evident that 0 ≤ Δ*_p_* ≤ 0.5. Using Equation (40) and setting *f_sp_* as in Equation (33), the estimation error can be expressed as

(53) $$\begin{array}{ll} {\hat{f}}_{d}-f_{d}=\delta_{f} & =\frac{{\bar{f}}_{A}{\tilde{\bar{S}}}_{A}+{\bar{f}}_{B}{\tilde{\bar{S}}}_{B}+{\bar{f}}_{C}{\tilde{\bar{S}}}_{C}}{{\tilde{\bar{S}}}_{A}+{\tilde{\bar{S}}}_{B}+{\tilde{\bar{S}}}_{C}}-f_{d}\\ & =\frac{(f_{d}+\Delta f_{A}){\tilde{\bar{S}}}_{A}+(f_{d}+\Delta f_{A}+f_{sp}){\tilde{\bar{S}}}_{B}+(f_{d}+\Delta f_{A}-f_{sp}){\tilde{\bar{S}}}_{C}}{{\tilde{\bar{S}}}_{A}+{\tilde{\bar{S}}}_{B}+{\tilde{\bar{S}}}_{C}}-f_{d}\\ & =\frac{\Delta f_{A}{\tilde{\bar{S}}}_{A}+(\Delta f_{A}+f_{sp}){\tilde{\bar{S}}}_{B}+(\Delta f_{A}-f_{sp}){\tilde{\bar{S}}}_{C}}{{\tilde{\bar{S}}}_{A}+{\tilde{\bar{S}}}_{B}+{\tilde{\bar{S}}}_{C}}\\ & =\Delta f_{A}+\frac{f_{sp}({\tilde{\bar{S}}}_{B}|-{\tilde{\bar{S}}}_{C})}{{\tilde{\bar{S}}}_{A}+{\tilde{\bar{S}}}_{B}+{\tilde{\bar{S}}}_{C}} \end{array}$$

From Equation (53), and using the definition Equation (52), we can calculate the expected value of *δ_f_* as

(54) $$E\{\delta_{f}\}=f_{sp}E\left\{\Delta_{p}+\frac{{\tilde{\bar{S}}}_{B}-{\tilde{\bar{S}}}_{C}}{{\tilde{\bar{S}}}_{A}+{\tilde{\bar{S}}}_{B}+{\tilde{\bar{S}}}_{C}}\right\}$$

From this result we observe that the residual error depends on both the noise contribution in the vector **S̅** and the parameter Δ*_p_*.

**Figure 12.**
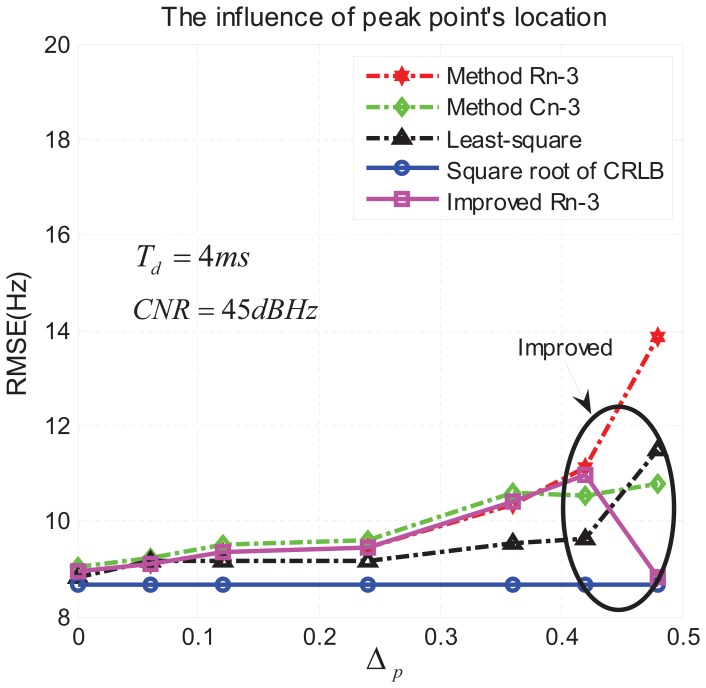
Influence of the quantization error on the RMSE as a function Δ*_p_*.

From Figure 12 we can see that Δ*_p_* influences the accuracy of the proposed three methods, especially when it comes to the top limit 0.5. This result suggested us to adopt a strategy to mitigate this effect. Recalling Figure 4, in which “A” is the peak point, “B” is the second high point and “C” is the lowest point independently from their relative position on the frequency axis, we can easily observe that, when Δ*_p_* is close to the limit 0.5, then *S̅_C_* is close to zero. In this case the residual error defined in Equation (39) introduces a significant error in the estimate Equation (40), since noise dominates in point “C”. This is experimentally proven in Figure 12, where the curves of R*_n_*-3 and C*_n_*-3 show an increasing RMSE as Δ*_p_* increases, while the LS appears slightly more robust.

So when Δ*_p_* is close to 0.5, to limit the accuracy degradation we changed the algorithm (40) as

(55) $${\hat{f}}_{d,\text{rel}}=\frac{{\bar{f}}_{A}{\tilde{\bar{S}}}_{A}+{\bar{f}}_{B}{\tilde{\bar{S}}}_{B}}{{\tilde{\bar{S}}}_{A}+{\tilde{\bar{S}}}_{B}}$$

which will be used whenever the point “C” gets close to zero. The idea is to ignore the “C” term, because when Δ*_p_* is close to 0.5, the true value S̅*_C_*is nearly zero and ${\tilde{\bar{S}}}_{C}$ mainly contains noise.

**Figure 13.**
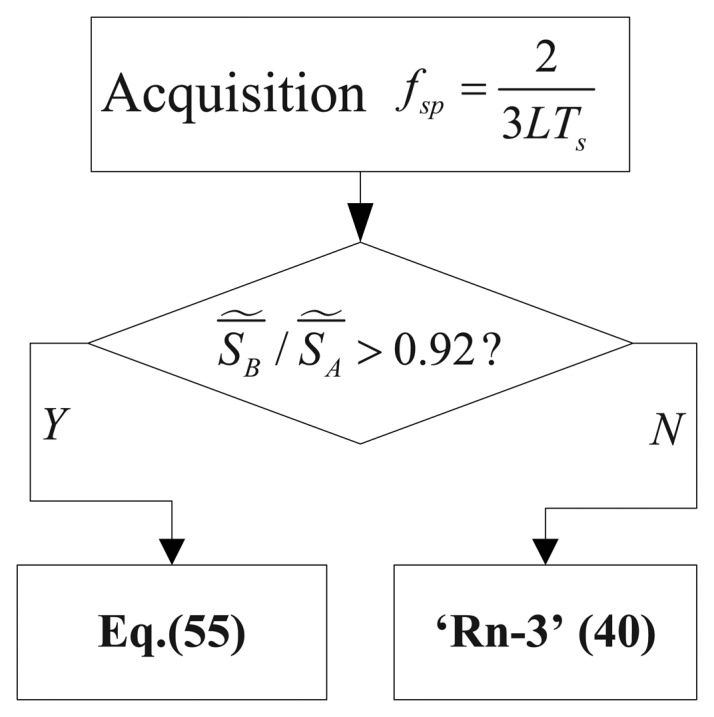
The strategy used in improved algorithm R*_n_*-3.

To implement this method, a threshold control has to be added, as drawn in Figure 13. Here we use the empirical criterion ${\tilde{\bar{S}}}_{B}/{\tilde{\bar{S}}}_{A}>0.92$ to decide whether Δp is critically close to 0.5 or not. In Figure 12 we can observe the result of the method drawn in Figure 13 (continuous line with square markers), which is able to consistently reduce the RMSE when Δp is close to 0.5.

### 5.2. The Influence of the Code Delay Error

The peak column selected in the search space (marked in Figure 4) also depends on the resolution of the search space in the code delay domain. The mentioned quantization error Δ*τ_q_* = *τ̅_p_* − *τ* affects the CAF samples with an amplitude scale factor |*R*(Δ*τ_q_*) | as shown in Equation (21), where now Δ*τ* = Δ*τ_q_*. This factor is expected to affect the performance of the estimators C*_n_*-3, and R*_n_*-3.

The influence of such a code delay error can be quantified by modifying the expression of the signal-to-noise ratio *ρ* in the CRLB Equation (49), so as to take into account the term *R*(Δ*τ_q_*). Thus, defining the modified $\text{SNR}\;\tilde{\rho }={\frac{A^{2}\left|R(\Delta \tau_{q})\right|}{2\sigma_{IF}^{2}}}^{2},$ Equation (49) becomes

(56) $$\mathrm{var}(\hat{f_{d}})\geq \frac{12}{{\left(2\pi \right)}^{2}\tilde{\rho }f_{s}T_{d}^{3}}$$

From Equation (56), because *R*(Δ*τ_q_* ≤ 1), we can conclude that the CRLB value increases as the code delay Δ*τ_q_* increases; this increase will be also experienced by the RMSE of the proposed algorithms. In conclusion the effect of the quantization error Δ*τ_q_* is an attenuation, which does not modify the results of Figures 8 and 9, except for a scaling factor in the abscissa.

## 6. Conclusions

In this paper a new family of algorithms is proposed for the fine estimation of the Doppler frequency based on an approximate analytical expression of the CAF The proposed methods have been analyzed in both ideal (*i.e.*, noiseless) and realistic (*i.e.*, noisy) scenarios and compared with a similar LS approach. The CRLB has been derived and used as benchmark for performance assessment. The influences of non-AWGN nuisances are also analyzed under a theoretical perspective. In application, from the experiments, we can see that the method R*_n_*-3 almost achieves the performance of LS, which is very close to CRLB, but the complexity of R*_n_*-3 is notably lower. Moreover, performance can be improved by adopting a simple averaging method.
